# BCG Vaccine Derived Peptides Induce SARS-CoV-2 T Cell Cross-Reactivity

**DOI:** 10.3389/fimmu.2021.692729

**Published:** 2021-08-05

**Authors:** Peter J. Eggenhuizen, Boaz H. Ng, Janet Chang, Ashleigh L. Fell, Rachel M. Y. Cheong, Wey Y. Wong, Poh-Yi Gan, Stephen R. Holdsworth, Joshua D. Ooi

**Affiliations:** ^1^Centre for Inflammatory Diseases, Department of Medicine, Monash Medical Centre, School of Clinical Sciences, Monash University, Clayton, VIC, Australia; ^2^Department of Immunology, Monash Health, Monash Medical Centre, Clayton, VIC, Australia

**Keywords:** COVID-19, BCG, heterologous immunity, cross-protection, T cell, vaccine

## Abstract

Epidemiological studies and clinical trials suggest Bacillus Calmette-Guérin (BCG) vaccine has protective effects against coronavirus disease 2019 (COVID-19). There are now over 30 clinical trials evaluating if BCG vaccination can prevent or reduce the severity of COVID-19. However, the mechanism by which BCG vaccination can induce severe acute respiratory syndrome coronavirus 2 (SARS-CoV-2)-specific T cell responses is unknown. Here, we identify 8 novel BCG-derived peptides with significant sequence homology to either SARS-CoV-2 NSP3 or NSP13-derived peptides. Using an *in vitro* co-culture system, we show that human CD4+ and CD8+ T cells primed with a BCG-derived peptide developed enhanced reactivity to its corresponding homologous SARS-CoV-2-derived peptide. As expected, HLA differences between individuals meant that not all persons developed immunogenic responses to all 8 BCG-derived peptides. Nevertheless, all of the 20 individuals that were primed with BCG-derived peptides developed enhanced T cell reactivity to at least 7 of 8 SARS-CoV-2-derived peptides. These findings provide an *in vitro* mechanism that may account, in part, for the epidemiologic observation that BCG vaccination confers some protection from COVID-19.

## Introduction

Severe acute respiratory syndrome coronavirus 2 (SARS-CoV-2) causes coronavirus disease 2019 (COVID-19) (1, 2). Alongside other immune cells, T cells are pivotal in mounting a successful immune response against COVID-19 as recovered individuals exhibit SARS-CoV-2 specific T cell memory, while T cell dysfunction and imbalance has been reported as a hallmark of severe COVID-19 (3, 4). Both CD4+ and CD8+ T cells have been implicated in COVID-19 with CD4+ T cells being broadly Th1-like by the secretion of cytokines interleukin-2 (IL-2), interferon gamma (IFN-*γ*) and tumor necrosis factor (TNF), and CD8+ T cells also secreting TNF and IFN-*γ* as well as effecting direct target cell lysis through the secretion of perforin and granzymes (5). Cross-reactive T cells between other human coronaviruses and SARS-CoV-2 have been identified, suggesting the potential role for T cell cross-protection in COVID-19 (6, 7). Here we investigated whether cross-reactive SARS-CoV-2-specific T cells can arise from Bacillus Calmette-Guérin (BCG)-derived peptide sensitization *in vitro*.

BCG vaccine containing live attenuated *Mycobacterium bovis*, hereafter referred to as BCG, typically vaccinates against tuberculosis (TB). BCG can also induce cross-protection against pathogens unrelated to TB through heterologous immunity, which although is generally not as effective as homologous immunity, may result in reduced disease severity or protective immunity (8). The cross-protective effects have shown to reduce all-cause mortality in children and respiratory tract infections in adults (9–12). One established mechanism of cross-protection is through BCG epigenetically modifying innate immune cells in the form of trained innate immunity lasting up to one year (13, 14). The heterologous effect of BCG vaccination on T cells has been demonstrated in other viral infections such as murine vaccinia virus and HPV papillomatosis (15–18).

Given the heterologous effects of BCG vaccination, there are over 30 clinical trials globally to test the cross-protective effect of BCG in COVID-19, notably the BRACE study involving 10,000 healthcare workers in Australia and the Netherlands (19). The majority of clinical trials addressing this are currently recruiting or ongoing. To date, at least 4 have concluded and shown BCG can provide some benefit in COVID-19 (20–23). Additionally, large country-level epidemiological analyses, as well as a study of > 6000 health care workers, have shown a negative correlation between BCG vaccination status, COVID-19 disease severity and SARS-CoV-2 IgG seroprevalence (24–27).

Here we show an *in vitro* mechanism whereby BCG-peptide-primed T cells have the capacity to cross-react with SARS-CoV-2-peptide homologues.

## Methods

### Patient Samples

Whole blood was collected from healthy donors with no prior known infection with TB or COVID-19 and who tested negative for serum IgG/IgM SARS-CoV-2 antibodies by SARS-CoV-2 Colloidal Gold Immunochromatography Assay Kit (MyBioSource). Donor characteristics are summarized in **Tables S2A, B**.

### Statistics

Data was analyzed using R Studio ver.1.3.959 and GraphPadPrism 7 (Graphpad Software Inc.). A Shapiro-Wilk test was used to determine normality followed by two-tailed, Wilcoxon-matched-pairs-signed-rank test to compare responses of BCG-primed with control-primed samples from responders. A two-tailed, Mann-Whitney test was used to compare responses of BCG vaccinated and BCG unvaccinated individuals.

### Study Approval

The study was conducted according to the Declaration of Helsinki; approved by Monash University HREC project ID 25834. Donors provided written informed consent.

### Data Reporting

No statistical methods were used to pre-determine sample size. The experiments were not randomized. The investigators were not blinded to allocation during experiments and assessment of outcomes.

### HLA Typing

Seven donors underwent high resolution class I and II molecular sequence-based typing performed by the Australian Red Cross Victorian Transplantation and Immunogenetics Service by next-generation sequencing. Three donors underwent low-resolution HLA-DR typing at the same provider. HLA typing results are contained within **Table S2B**.

### HLA Binding Prediction and Allele Coverage

Global allele coverage of HLA-typed donors was assessed at IEDB Analysis Resource – Population Coverage (28). NetMHCpan-4.1 and NetMHCIIpan-4.0 were used to predict binding affinity of homologous peptides to a globally representative collection of MHCI or MHCII alleles plus the alleles of our HLA-typed donors using artificial neural networks (29–31). For each region of homology, 9mers for MHCI and 15mers for MHCII overlapping by 1 amino acid underwent affinity analysis. Affinity rank was generated that normalizes prediction score by comparing to prediction of a set of random peptides. An affinity rank score of < 2 was called a strong binder. An affinity rank score of ≥ 2 and ≤ 10 was called a binder. An affinity score of > 10 was called a non-binder.

### Sequence Alignment

Protein BLAST search of the SARS-CoV-2 proteome (sequence ID NC_045512.2) restricted to *Mycobacterium bovis* (BCG) was performed using the NCBI blastp suite (https://blast.ncbi.nlm.nih.gov/Blast.cgi). Protein sequences from SARS-CoV-2 NSP3 (YP_009725299.1), SARS CoV-2 NSP13 (YP_009725308.1), BCG RecB nuclease (KAF3412556.1), BCG UPF0189 protein (AHM07651.1), BCG Macro domain containing protein (WP_003909539.1), BCG zinc metalloprotease FtsH (AMC52863.1) and human CLIP (NP_001020330.1) were obtained from the NCBI Database (https://www.ncbi.nlm.nih.gov/protein/). Sequence alignment of SARS-CoV-2 and BCG homologues was performed using EMBOSS Needle Pairwise Sequence Alignment (32).

### Peptides

15mer peptides were synthesized with an N-terminal free amine (H-) and a free acid group at the C-terminus (-OH). Peptides were ≥ 90% pure as assessed by reversed-phase high-performance liquid chromatography (RP-HPLC) (Mimotopes). Peptide sequences used in this study can be found in **Table S1** and control peptide CLIP_103-117_ (PVSKMRMATPLLMQA). Lyophilized peptide was reconstituted in sterile MilliQ water with 5% (v/v) DMSO (Sigma). Final concentration of peptides used in culture was 10µg/mL and final concentration of DMSO in the cultures was 0.005% (v/v).

### Monocyte Derived DC Production

Human PBMCs were freshly isolated from whole donor blood in K2EDTA anticoagulant Vacutainers (BD) using Lymphoprep density gradient medium (Stemcell) and SepMate tubes (Stemcell). PBMCs were enumerated in a haemocytometer with trypan blue 0.4% (Sigma) and the CD14+ CD16- monocytes were then magnetically separated using EasySep Human Monocyte Isolation Kit and EasySep Magnet following manufacturer’s instructions (Stemcell). Freshly isolated monocytes were then enumerated in a haemocytometer with 0.4% trypan blue and if viability was ≥90%, differentiation culture was established to differentiate the monocytes into dendritic cells using ImmunoCult Dendritic Cell Culture Kit following instructions of the manufacturer (Stemcell). According to the protocol (Stemcell), immature DCs used in the intracellular cytokine staining (ICS) co-culture did not receive maturation supplement on day 5 of culture and mature DCs used in the proliferation and memory co-culture received maturation supplement on day 5 of culture. After 7 days culture, immature DCs were used for the ICS co-culture and mature DCs were used for the proliferation co-culture.

### T Cell Isolation

Human CD3+ T cells were isolated from fresh whole donor blood in K2EDTA tubes using RosetteSep HLA T Cell Enrichment Cocktail according to instructions of the manufacturer (Stemcell). Isolated CD3+ T cells were enumerated in a haemocytometer with 0.4% trypan blue (Sigma) and only used when viability was ≥90%. CD3+ T cells were then used in the ICS and proliferation co-cultures.

### ICS Co-Culture Setup

**Figure 1:**
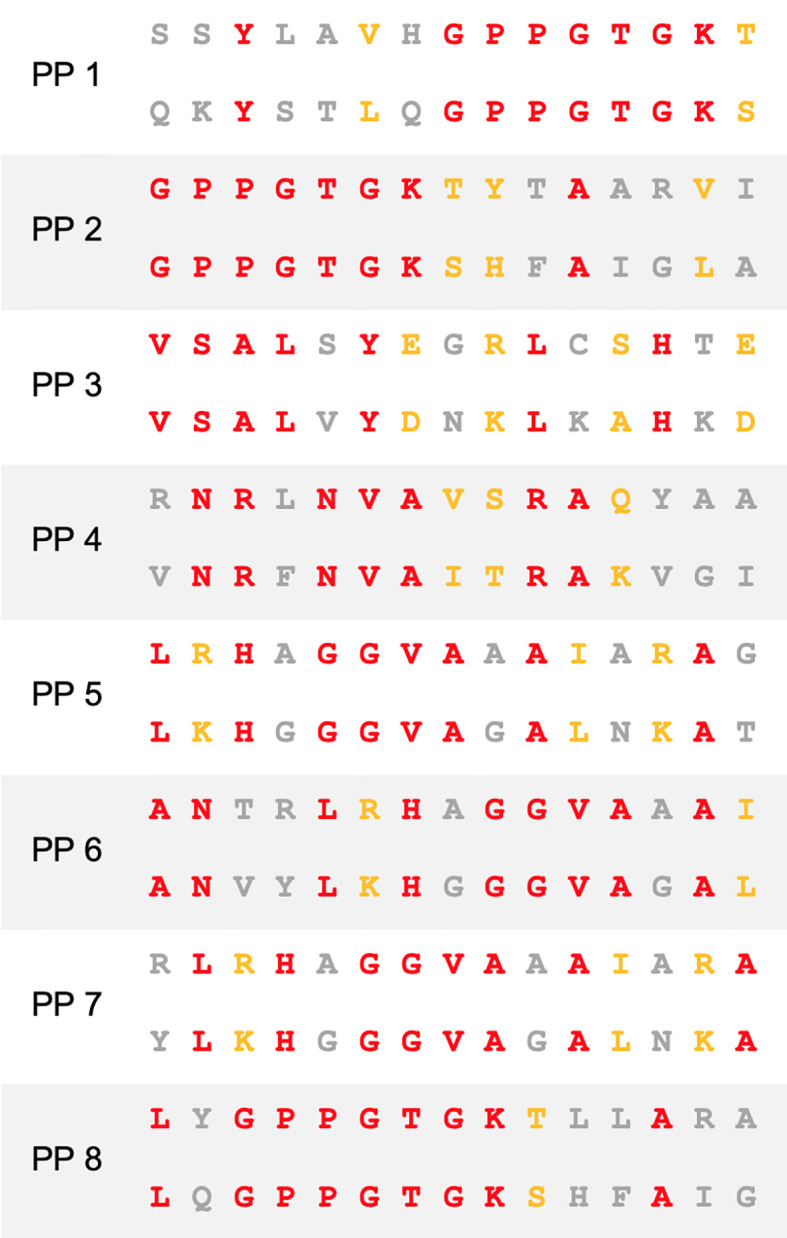
Sequence homology between BCG and SARS-CoV-2. Amino acid sequence alignment of peptide pairs (PP) of BCG (top) and SARS-CoV-2 (bottom) used in this study. Red–identity. Yellow–similarity. Grey–no identity/similarity.

An ICS co-culture assay to assess T cell cross-reactivity was adapted from previously published methods for the measurement of T cell cross-reactivity (33). Specifically, ICS co-culture was initiated with 100,000 freshly isolated human CD3+ T cells, 10,000 human immature monocyte-derived DCs and 10µg/mL of BCG peptide from PP1-8 (**Figure 1**) or control peptide (PVSKMRMATPLLMQA) in a 96 well round-bottom plate (Corning) at 100µL per well of complete RPMI (Gibco) supplemented with 10% autologous human serum, 100 U/mL penicillin and 0.1 mg/mL streptomycin (Gibco), 2mM L-glutamine (Gibco) and 50µM 2-mercaptoethanol (Sigma). Positive assay control received anti-human CD2, anti-human CD3, and anti-human CD28 coated MACS iBeads at a ratio of 1 bead:2cells prepared from the human T cell activation/expansion kit as per the manufacturer’s instructions (Miltenyi). Negative assay control received no peptides. Co-culture was incubated at 37°C in a CO_2_ incubator (Binder). Five days later, the co-culture was supplemented with 40IU/mL recombinant human IL-2 (Stemcell) and reincubated. On day 7 of co-culture, cells were rested by washing twice in 250µL PBS to remove peptides and resuspended in 100µL fresh complete RPMI formulated as above with no peptides and reincubated. On day 9 of co-culture, cells were restimulated by washing twice with 250µL PBS then 10,000 freshly-cultured, immature DCs were added per well with 10µg/mL of SARS-CoV-2 peptide from PP1-8 (**Figure 1**) or control peptide (PVSKMRMATPLLMQA) and 1µg/mL anti-human CD28 monoclonal antibody (clone CD28.2, eBioscience) in serum-free RPMI. Positive assay control received anti-human CD2, anti-human CD3, and anti-human CD28 coated MACS iBeads at a ratio of 1 bead:2cells. Negative assay control received no peptides. To pulse the DCs with peptide, co-culture was incubated for 2 hours at 37°C in a CO_2_ incubator (Binder). After 2 hours, media was adjusted to contain 10% autologous serum and 1X protein transport inhibitor cocktail containing brefeldin A and monensin (eBioscience) was added and reincubated. After 6 hours at 37°C in a CO_2_ incubator, cells were harvested for flow cytometric analysis by ICS. The entire culture system was setup to be autologous.

HLA blocking was performed on selected HLA-typed donor samples as per the aforementioned ICS co-culture setup with the additional step of adding the respective blocking antibody on day 9. Blocking antibodies mouse anti-human HLA-DR (clone L243, Abcam), mouse anti-human HLA-DQ (clone SPV-L3, Novus Biologicals), mouse anti-human HLA-A/B/C (clone W6/32, eBioscience) and isotype control mouse IgG2a kappa (clone eBM2a, eBioscience) were incubated at 10µg/mL with immature DCs for 1hr at 37°C in a CO_2_ incubator prior to addition of peptide and T cells.

Comparison of BCG vaccinated and unvaccinated individuals was performed as per the aforementioned ICS co-culture setup and instead performing only one stimulation using the SARS-CoV-2 peptide.

### Proliferation Co-Culture Setup

Proliferation co-culture was initiated with 100,000 freshly isolated CD3+ T cells stained with cell proliferation dye Cell Trace Yellow (CTY) according to the manufacturer (Invitrogen), 10,000 human mature DCs and 10µg/mL of BCG peptide from PP1-8 (**Figure 1**) or control peptide (PVSKMRMATPLLMQA) in a 96 well round-bottom plate (Corning) at 100µL per well of complete RPMI (Gibco) supplemented with 10% autologous human serum, 100 U/mL penicillin and 0.1 mg/mL streptomycin (Gibco), 2mM L-glutamine (Gibco) and 50µM 2-mercaptoethanol (Sigma). Positive assay control received anti-human CD2, anti-human CD3, and anti-human CD28 coated MACS iBeads at a ratio of 1 bead:2cells prepared from the human T cell activation/expansion kit as per the manufacturer’s instructions (Miltenyi). Negative assay control received no peptides. Co-culture was incubated at 37°C in a CO_2_ incubator (Binder). Seven days later, cells were washed twice in 250uL PBS to remove peptides and resuspended in 100µL complete RPMI formulated as above with no peptides and reincubated. On day 9 of co-culture, cells were washed twice in 250µL PBS and stained with Cell Trace Violet (CTV) cell proliferation dye according to the manufacturer’s instructions (Invitrogen). Then 10,000 freshly-cultured, human mature DCs were added per well with 10µg/mL of SARS-CoV-2 peptide from PP1-8 (**Figure 1**) or control peptide (PVSKMRMATPLLMQA). Positive assay control received anti-human CD2, anti-human CD3, and anti-human CD28 coated MACS iBeads at a ratio of 1 bead:2cells. Negative assay control received no peptides. Co-culture was incubated at 37°C in a CO_2_ incubator for 7 days then harvested for flow cytometric analysis. The entire culture system was setup to be autologous.

### ICS Flow Cytometry Staining and Analysis

**Figure 2:**
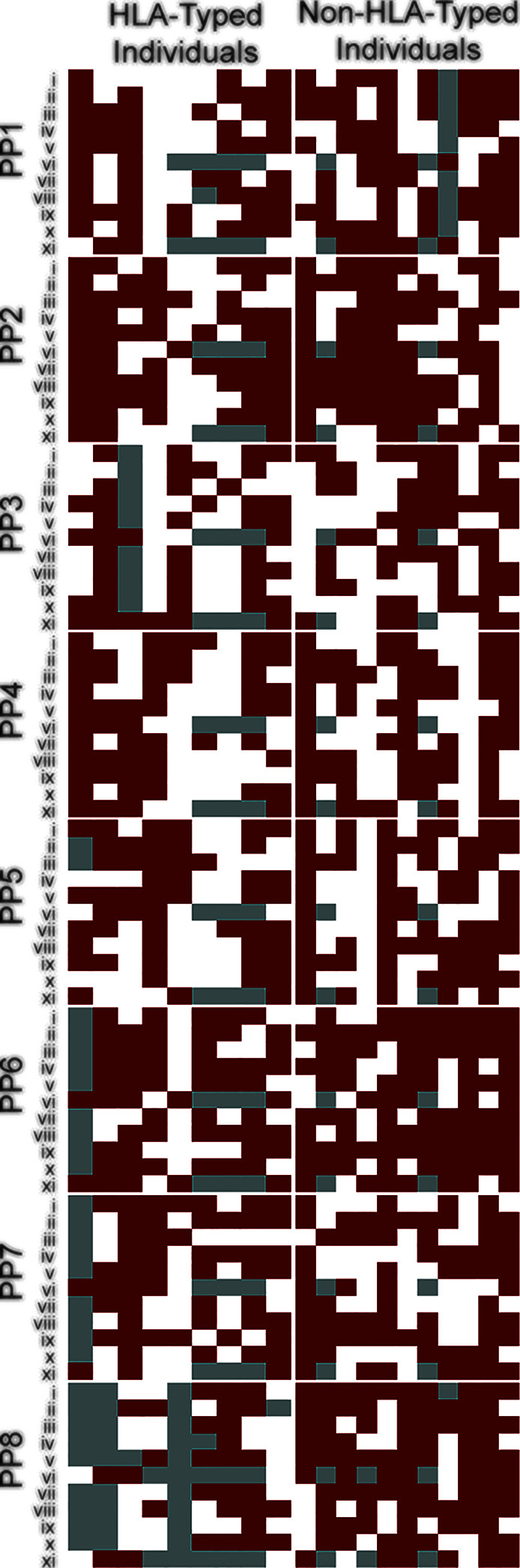
BCG induces broad cross-reactive T cell responses across individuals. Heat map of individuals representing global HLA coverage shows improved SARS-CoV-2 T cell responses when pre-stimulated with BCG peptide. Individual donor T cell responses to the 8 peptide pairs (PP1-PP8) across 11 parameters (i-xi) determined by flow cytometry. i–CD8+IFN-*γ*, ii–CD8+TNF, iii–CD8+IL-2, iv–CD8+CD69, v–CD8+Perforin, vi–CD8+proliferation, vii–CD4+IFN-*γ*, viii–CD4+TNF, ix–CD4+IL-2, x–CD4+CD69, xi–CD4+proliferation. A responder (red) is defined as showing a positive response after subtraction of the control-primed response to SARS-CoV-2. A non-responder (white) is defined as showing no positive staining after subtraction of the control response. Grey –no data. Individuals were grouped by known or unknown HLA-type highlighting similar patterns between them.

**Figure 3:**
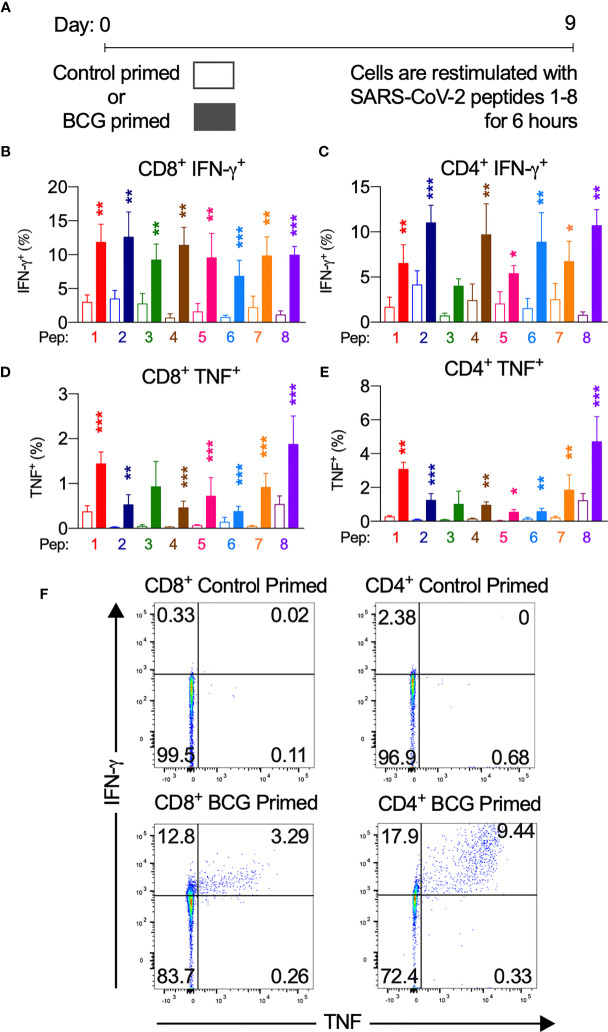
BCG priming enhances SARS-CoV-2 T cell responses. BCG-peptide-primed T cells restimulated with SARS-CoV-2-peptide-pulsed DCs analysed by flow cytometry. Unshaded bars- Control primed (irrelevant peptide, PVSKMRMATPLLMQA), then SARS-CoV-2-peptide1-8 restimulated. Shaded bars– BCG peptide 1-8 primed then SARS-CoV-2-peptide homologue restimulated. **(A)** Brief culture timeline, **(B)** CD8+IFN-*γ*+ responses (*n* = 9-12), **(C)** CD4+IFN-*γ*+ responses (*n* = 5-13), **(D)** CD8+TNF+ responses (*n* = 4-14), **(E)** CD4+TNF+ responses (*n* = 6-16), **(F)** Representative TNF (x-axis) and IFN-*γ* (y-axis) dot plots of a responder donor with their corresponding SARS-CoV-2 primary response control. *P < 0.05, **P < 0.01,***P < 0.001 by Wilcoxon-matched-pairs-signed-rank test, comparing magnitude of response to SARS-CoV-2 peptides with or without BCG priming.

After culturing, cells were stained with Live/Dead Fixable Near Infra-Red Dead Cell Stain Kit according to the manufacturer’s instructions (Invitrogen). Cells were then stained with surface markers anti-human CD3 Brilliant Violet 510 (clone OKT3, Biolegend), anti-human CD4 APC (clone OKT4, eBioscience), anti-human CD8 Alexa Fluor 488 (clone HIT8a, Biolegend) and anti-human CD69 Brilliant UV 395 (clone FN50, BD). After surface staining, cells were fixed and permeabilized with Transcription Factor Staining Buffer Set according to the manufacturer’s instructions (eBioscience). Cells were subsequently stained for intracellular markers with anti-human IFN-*γ* PE Cy7 (clone 4S.B3, eBioscience), anti-human TNF Brilliant Violet 421 (clone Mab11, Biolegend), anti-human IL-2 Brilliant Blue 700 (clone MQ1-17H12, BD) and anti-human perforin PE (clone B-D48, Biolegend). Single color controls were prepared using UltraComp eBeads (Invitrogen) for single color control antibodies and ArC amine reactive compensation bead kit (Invitrogen) for Live/Dead single color control. After staining, cells were resuspended in PBS and acquired on an LSR-Fortessa X20 flow cytometer (BD) using BD FACSDiva software version 8.0.1. Samples were analyzed in FlowJo 10.6.2. FMO controls were used to determine positive gating (**Figure S3**). Individuals that responded in the given parameters to SARS-CoV-2 after BCG priming (**Figure 2**) were defined as showing positive staining after subtraction of the primary SARS-CoV-2 response control (control peptide-primed, SARS-CoV-2 peptide stimulated). A non-responder was defined as showing no positive staining after subtraction of the primary SARS-CoV-2 response control. For statistical analysis (**Figure 3** and **Figures S5, S6**), the responders were selected as those with positive staining after subtraction of the primary SARS-CoV-2 response control or restimulation background control (BCG primed, irrelevant peptide restimulated). The responders then had the restimulation background control (BCG primed and irrelevant peptide restimulated) subtracted from the corresponding BCG primed, SARS-CoV-2 test sample and the primary SARS-CoV-2 response control to remove any assay related background stimulation.

### Proliferation Flow Cytometry Staining and Analysis

**Figure 4:**
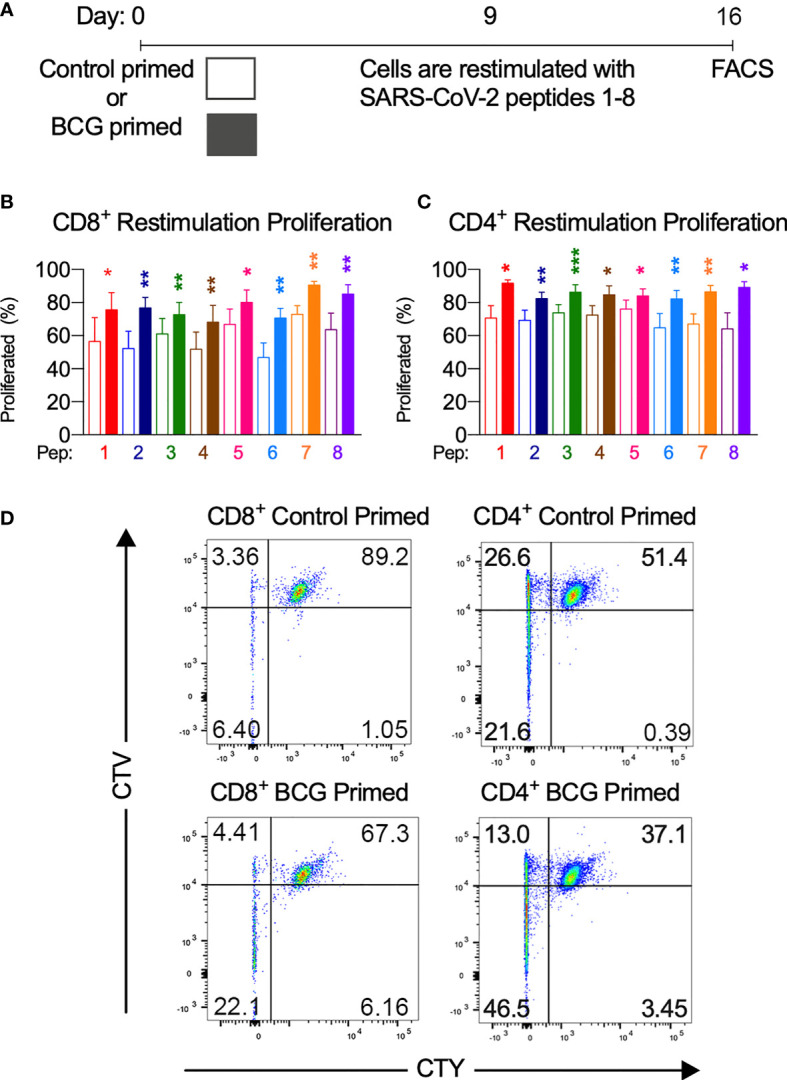
BCG priming enhances T cell proliferation. **(A)** Brief culture timeline. **(B)** CD8+ restimulation proliferation is enhanced by BCG priming (*n*=9-12). **(C)** CD4+ restimulation proliferation is enhanced by BCG priming (*n*=6-11). Unshaded bars– Control-primed (irrelevant peptide, PVSKMRMATPLLMQA), then SARS-CoV-2-peptide restimulated. Shaded bars– BCG peptide primed then SARS-CoV-2-peptide homologue restimulated. **(D)** Representative CTV *vs.* CTY dot plots of CD4+ and CD8+ cultured cells indicating proliferation. Top right quadrant gate (CTY^hi^CTV^hi^ cells) did not proliferate upon priming or restimulation. Top left quadrant gate (CTY^lo^CTV^hi^ cells) proliferated upon priming but not with restimulation. Bottom left quadrant gate (CTY^lo^CTV^lo^ cells) proliferated upon both priming and restimulation. *P < 0.05, **P < 0.01, ***P < 0.001 by Wilcoxon-matched-pairs-signed rank test, comparing the magnitude of response to SARS-CoV-2 peptides with or without BCG priming.

After culturing, cells were stained with Live/Dead Fixable Near Infra-Red Dead Cell Stain Kit according to the manufacturer’s instructions. Cells were then stained with surface markers anti-human CD3 PerCP (clone SK7, Biolegend), anti-human CD4 APC (clone OKT4, eBioscience), and anti-human CD8 Alexa Fluor 488 (clone HIT8a, Biolegend). Single color controls were prepared using UltraComp eBeads (Invitrogen) for single color control antibodies, ArC amine reactive compensation bead kit (Invitrogen) for Live/Dead single color control and Cell Trace Violet and Cell Trace Yellow single stained co-cultured cells along with unstained co-cultured cells. After staining, cells were resuspended in 0.5% BSA, 2mM EDTA/PBS and acquired on an LSR-Fortessa X20 flow cytometer (BD) using BD FACSDiva software version 8.0.1.fcs files were analyzed in FlowJo 10.6.2. All fluorescence based gating except CTY, and CTV is determined based on fluorescence minus one (FMO) controls (**Figure S4**). CTV and CTY gating are based on the point at which the first cell division took place visible by fluorescence dye dilution. Individuals that showed a proliferation response when BCG primed, SARS-CoV-2 restimulated (**Figure 2**) were defined as showing positive staining after subtraction of the primary SARS-CoV-2 response control (irrelevant peptide primed, SARS-CoV-2 peptide stimulated). A non-responder was defined as showing no positive staining after subtraction of the primary SARS-CoV-2 response control. For statistical analysis (**Figure 4**), the responders were selected as those with positive staining after subtraction of the primary SARS-CoV-2 response control or restimulation background control (BCG primed, irrelevant peptide restimulated). Proliferation in response to BCG priming and SARS-CoV-2 restimulation was calculated as the proportion of CD4+ or CD8+ cells that underwent proliferation post-priming and post-restimulation (CTY^lo^CTV^lo^) of total proliferated cells (CTY^lo^CTV^lo^ and CTY^lo^ CTV^high^).

## Results

### SARS-CoV-2 Amino Acid Homology With BCG

T cells specific for SARS-CoV-2 are well recognized as pivotal in mounting a successful immune response to COVID-19 (5). To determine whether homology exists between BCG and SARS-CoV-2, we first performed NCBI Protein Blast searches against the SARS-CoV-2 proteome, restricting results to BCG proteins. Regions of protein sequence homology were identified between BCG sequences and the non-structural proteins NSP3 and NSP13 located in ORF1ab of SARS-CoV-2 (**Figure 1** and **Table S1**). When processed as 15mers for MHCII presentation, these regions exhibit up to 60% identity and 73.3% similarity between BCG and SARS-CoV-2 (**Table S1A**). Percent identity and similarity of constituent 9mers for MHCI presentation are up to 88.8% and 100%, respectively (**Table S1B**).

To assess the capacity of BCG epitopes to bind HLA alleles that broadly cover the global population, we performed *in silico* prediction analyses of peptide-MHC binding affinity using NetMHCIIpan 4.0 and NetMHCpan 4.1 across each region of homology as 9mers or 15mers overlapping by 1 amino acid residue in MHCI and MHCII binding, respectively (29). HLA alleles in the analysis were selected based on previously reported reference sets giving maximal global population coverage (28, 30). We found that the BCG derived peptides with homologous sequences to SARS-CoV-2 peptides exhibited broad MHC class II and MHC I binding capacity (**Figures S1, S2**).

To determine the cross-reactive immunogenicity of these BCG derived peptides across diverse HLA-types, we selected 10 healthy HLA-typed blood donors with different HLA types (**Table S2B**). Based on IEDB population coverage, our collection of HLA-typed individuals gave a global MHC class I and II coverage of 97.21% and 99.97%, respectively (34). In addition, binding affinity predictions of the homologous peptides to HLA alleles from the 10 HLA-typed donors used in this study were analyzed (**Figures S1, S2**). To determine if HLA-typing was necessary, we also tested the cross-reactive immunogenicity of the BCG derived peptides on 10 non-HLA-typed persons. Based on high homology and strong binding, a selection of eight different 15mer peptide pairs (PP1-8) were chosen for subsequent experimentation on human donors (**Figure 1**). The 15mers are of an appropriate length to be presented by MHC class II to CD4+ T cells. Each 15mer contained within it 7 different 9mers (**Table S1B**), which, after antigen processing in immature dendritic cells (DCs), can be presented to CD8+ T cells *via* MHC class I.

### T Cell Cross-Reactivity

To determine if priming with BCG peptide enhances T cell responses to SARS-CoV-2 peptides, we compared CD4+ and CD8+ T cell responses to SARS-CoV2 peptides using cells that were either primed with irrelevant control peptide (PVSKMRMATPLLMQA) or BCG peptide. CD3+ T cells were isolated from donors (*n*=20, **Table S2A**) and co-cultured with DCs *in vitro*. Individual BCG peptides were first used to sensitize and expand the BCG-specific T cells, simulating a BCG vaccination. T cells were then rested for two days without antigen stimulation then re-stimulated with corresponding homologous SARS-CoV-2 peptides. To measure T cell responses, we performed intracellular cytokine staining (ICS) for IFN-*γ*, TNF, IL-2, perforin; surface staining for the early T cell activation marker CD69, and a two-color proliferation assay to differentiate between a primary proliferative and secondary proliferative response. A positive response was defined as an increase compared to control. All individuals (*n*=20) exhibited a positive response to at least 7 out of 8 SARS-CoV-2 peptides (**Figure 2**).

Next, we assessed the degree of SARS-CoV-2 T cell reactivity enhancement conferred by BCG priming compared to control primed T cells (**Figure 3**, and **Figures S5, S6**). In CD8+ cytotoxic T cells, IFN-*γ*, TNF and IL-2 cytokine production across at least 7 of the 8 peptide pairs significantly increased (**Figure 3** and **Figure S5A**). IFN-*γ* mean fold increase in expression ranged from 2.3-fold (PP3) to 16.3-fold (PP4). Mean fold increase in TNF expression from CD8+ cells ranged from 1.7-fold (PP6) to 23.9-fold (PP2). IL-2 production from CD8+ cells showed a mean fold increase from 3.1-fold (PP2) to 33.1-fold (PP5).

CD4+ T helper cells exhibited similarly significantly increased IFN-*γ*, TNF and IL-2 production across at least 7 of the 8 peptide pairs (**Figure 3** and **Figure S6A**). In CD4+ cells from responder individuals, IFN-*γ* mean fold increase in expression ranged from 1.7-fold (PP2, PP5 & PP7) to 12.9-fold (PP8). Mean fold increase in TNF from CD4+ cells ranged from 2.9-fold (PP8) to 14.1-fold (PP2 & PP5). IL-2 production from CD4+ cells showed a mean fold increase from 2.1-fold (PP5) to 12.3-fold (PP8).

To confirm that the observed cross-reactive T cell responses arise from peptide-MHC interaction with the T cell, we blocked HLA and measured T cell cytokine responses, as above. We identified 15mers and 9mers for CD4+ and CD8+ responses, respectively that were *in silico* predicted to bind to one HLA allele only out of all HLA alleles of a HLA-typed individual. We then selectively blocked this response using a blocking antibody against that particular HLA (either anti-HLA-DR or anti-HLA-DQ for MHC class II blocking of CD4+ T cell responses and anti-HLA-A/B/C for MHC class I blocking of CD8+ T cell responses). Without HLA blocking or blocking with isotype control antibody, T cell IFN-*γ* and TNF were predictably high. Upon HLA-specific blocking, there was a near complete abrogation of cytokine responses indicating that the T cell restimulation responses arise from cross-reactive T cell receptor binding to peptide-MHC on the DCs (**Figure S8**).

Since the COVID-19 CD8+ response involves the secretion of perforin and granzymes for an effective antiviral response, we measured perforin expression by ICS. We found that CD8+ T cells primed with BCG-derived peptides had an enhanced perforin expression upon SARS-CoV-2 restimulation when compared to control primed cells (**Figure S5B**). Cross-reactive perforin expression in responders was significantly increased across all 8 peptide pairs with a mean fold-increase ranging from 1.9-fold (PP1) to 47.2-fold (PP4).

In order to mount an effective T cell response to COVID-19, antigen-specific T cells must become activated and undergo clonal expansion. To assess whether T cells pre-stimulated with BCG-derived peptides exhibit enhanced T cell activation when restimulated with SARS-CoV-2 homologues, expression of early T cell activation marker CD69 was assessed by flow cytometry. We show that when compared to a SARS-CoV-2 primary response, the BCG primed T cells increased CD69 expression across 7 of the 8 peptide pairs in CD4+ and CD8+ T cells (**Figures S5C, S6B**). CD69 expression in responders showed a mean fold-increase ranging from 3.2-fold (PP1) to 29.6-fold (PP5) for CD4+ cells and from 1.7-fold (PP1) to 10.5-fold (PP2) for CD8+ cells.

To assess whether T cells primed with BCG derived peptides show enhanced T cell proliferation upon SARS-CoV-2 peptide restimulation, cell proliferation dye cell trace yellow (CTY) was used to assess the proliferation after BCG priming followed by cell trace violet (CTV) to assess the proliferation after SARS-CoV-2 restimulation. All donor samples primed with BCG peptide developed enhanced T cell proliferation to at least 3 out of the 8 SARS-CoV-2 peptides tested (**Figure 2**). The magnitude of the enhanced proliferative response was also assessed in BCG-primed individuals who responded SARS-CoV-2 restimulation. Specifically, we compared the SARS-CoV-2 peptide induced proliferation in cells that were first sensitized with BCG peptide or with control peptide. In all of the tested peptide pairs (PP1-PP8) and across both CD4+ and CD8+ T cells, BCG peptide sensitized cells developed significantly enhanced proliferation to its SARS-CoV-2 homologous peptide (**Figure 4**). In the responders, T cell proliferation was enhanced in CD8+ T cells between 19% (PP3 and PP5) to 51% (PP6) and in CD4+ T cells by 11% (PP5) to 39% (PP8).

We analyzed an equal number of males and females in this study (*n*=10 each) and no significant sex-specific differences were found in the parameters measured.

To compare SARS-CoV-2 peptide T cell responses between BCG-vaccinated and BCG-unvaccinated individuals, CD3+ T cell and immature DC co-cultures were pulsed with one of the 8 SARS-CoV-2 peptides (**Figure 1**) for 6hr and IFN-*γ* and TNF responses were measured by ICS (**Figure S9**). Across all 8 SARS-CoV-2 peptides and across the four tested parameters (CD8^+^ IFN-*γ*, CD8 TNF^+^, CD4 IFN-*γ* and CD4^+^ TNF), a trend was observed for increased SARS-CoV-2 peptide-induced CD4^+^ and CD8^+^ T cell responses in BCG vaccinated individuals compared to unvaccinated. However, this trend only reached significance in 3 parameters, namely peptide 5 on TNF production in CD8+ cell, peptide 3 on TNF production in CD4+ cells and peptide 8 on IFN-*γ* production in CD4+ cells.

## Discussion

T cell-dependent heterologous immunity can occur in a number of different ways, which can be broadly summarized as either TCR-dependent or TCR-independent. TCR-independent heterologous immunity can arise through nonspecific activation by virus-induced cytokines such as IL-12 and IL-18 without TCR involvement whereby the second infection stimulates memory T cells from the first infection (35, 36). TCR-dependent heterologous immunity can arise from direct cross-reactivity between unrelated pathogens, whereby memory T cells generated by the first infection/immunization cross-react with antigens from the second infection (37). It is this instance of TCR-dependent heterologous immunity which we propose as a potential mechanism for cross-reactivity between BCG vaccination and SARS-CoV-2 infection.

Similar epitopes shared between BCG and SARS-CoV-2 have recently been identified as having the potential for cross-reactive adaptive immunity (38). We identified 8 novel protein sequences with significant homology between BCG and NSP3 and NSP13 of SARS-CoV-2. NSP3 is a papain-like proteinase that shares a macro-domain with BCG proteins: macro-domain-containing protein and UPF0189 protein. This macro-domain-containing protein is conserved among the *Mycobacterium tuberculosis* complex including BCG (accession number WP_003909539.1), which encompasses strains used in BCG vaccine worldwide such as Pasteur and Connaught. NSP13 is a helicase that shares homology with BCG proteins RecB nuclease and zinc-metalloprotease-FtsH. Both RecB nuclease and zinc-metalloprotease-FtsH contain a walker-A-motif sequence that is identical in NSP13 of SARS-CoV-2. Additionally, RecB nuclease contains two other regions of homology with NSP13 around amino acid residues 952-966 and 1093-1107. As previously reported, NSP13 is highly conserved between other human coronaviruses. Thus, the T cell cross-protective potential of BCG may exist not only for SARS-CoV-2 but potentially with other human coronaviruses that cause the common cold (229E, NL63, OC43 and HKU1) and the more serious human coronaviruses SARS-CoV and Middle East respiratory syndrome coronavirus (MERS-CoV). NSP3 is, however, not as widely conserved among coronaviruses (6, 39).

For T cells to respond to antigen homologues, a significant degree of homology must also be paired with the capacity of an immunogenic peptide to bind cognate MHC class I or II. In the context of COVID-19, HLA binding has been reported as important in COVID-19 severity. Patients with mild COVID-19 presented MHCI molecules with a higher theoretical affinity than those with moderate to severe COVID-19 (31). We showed through *in silico* binding prediction assays that the BCG derived peptides with homologous sequences to SARS-CoV-2 peptides exhibited broad MHC class I and II binding capacity permitting antigen presentation across a globally representative HLA pool (**Figures S1, S2**). From these peptides, a selection of 8 homologue pairs were selected for *in vitro* co-culture analysis based on both high homology and high and broad peptide-HLA affinity (**Figure 1**).

The T cell responses measured were IFN-*γ*, TNF, IL-2, perforin and surface staining for the early T cell activation marker CD69, as well as a two-color proliferation assay to differentiate between a primary proliferative and secondary proliferative response. The enhanced cross-reactive response in BCG-primed, SARS-CoV-2 restimulated compared to not BCG-primed (**Figure 2**) confirms the high and broad HLA binding affinity prediction. We also show these cross-reactive peptides are immunogenic as they can elicit CD4+ Th1-like responses and robust CD8+ responses upon SARS-CoV-2 restimulation.

IFN-*γ*; TNF and IL-2 cytokine responses were significantly increased across at least 7 of the 8 peptide pairs tested in both CD4+ and CD8+ T cells. Patterns of cytokine production were varied between individuals and peptide pairs, which is reflected in the complex pattern of T cell cytokine expression and phenotypes that BCG vaccination is known to produce (40). Indeed, the IFN-*γ* and TNF response was mixed with some individuals making only IFN-*γ* or TNF in response to a particular peptide pair and some being positive for both (**Figure S7**). This observation is concordant with previously reported responses in COVID-19 (5, 6).

In CD8+ T cells, the target cell lysis protein, perforin, was significantly increased in the BCG-primed group compared to control-primed across all 8 peptide pairs (**Figure S5B**). The heightened perforin response suggests cross-reactive CD8+ T cells have the capacity to affect a superior antiviral response by target cell lysis. CD4+ and CD8+ T cell activation and proliferation were measured by activation marker CD69 expression and cell proliferation dye dilution. In at least 7 of the 8 tested peptide pairs across CD4+ and CD8+ T cells, BCG peptide sensitized cells developed significantly enhanced activation and proliferation to its SARS-CoV-2 homologous peptide (**Figures S5C**, **S6B**). The increase in T cell activation permits clonal expansion of the cross-reactive T cells and the development of their effector functions, which is necessary in mounting an effective immune response against SARS-CoV-2.

HLA-blocking experiments showed the T cell cytokine responses were abrogated when the HLA was blocked (**Figure S8**). This indicates the mechanism is the result of TCR-dependent heterologous immunity whereby BCG-primed, SARS-CoV-2 restimulation responses arise from peptide-MHC on the DCs binding to a cross-reactive T cell receptor on CD4+ or CD8+ T cells.

Assessing the 11 tested parameters in this study across both CD4^+^ and CD8^+^ T cells, it can be seen that all 8 identified peptides are capable of eliciting significant T cell cross-reactivity across a broad range of HLA-typed individuals. Therefore, instead of identifying one dominant epitope, a set of 8 epitopes were identified. Further characterization and delineation of these cross-reactive epitopes will assist in deeper understanding of this mechanism such as by using alanine substitution assays to determine the dominant amino acids at play here and antigen-specific tetramers to quantify the natural frequency of cross-reactive T cells. The 8 epitopes capable of T cell cross-reactivity would likely have an additive effect when administered altogether such as in a BCG vaccine.

A comparison of the SARS-CoV-2 peptide immune response in COVID-19 unaffected individuals either vaccinated with BCG or never vaccinated with BCG showed the BCG vaccinated individuals exhibited across all peptides a trend to increased TNF and IFN-*γ* responses in CD4^+^ and CD8^+^ T cells and three of these parameters were statistically significant (**Figure S9**). Despite the lack of statistical significance, this is still a noteworthy result since the BCG vaccinated group were all adults who received their BCG vaccination decades earlier during childhood. Further studies are needed to assess whether a recent BCG vaccination or booster, such as those being administered in the clinical trials, would enhance cross-reactive T cells. Another case for consideration is the low frequency of memory T cells present in peripheral blood compared to other sites such as the spleen and lymph nodes, which may restrict the ability to properly identify and characterize the cross-reactive memory T cells. The low number of cells assayed also further restricted the ability to assess the presence of these antigen-specific T cells.

Further research into the clinical significance of such cross-reactive T cells is critical to characterize any protective benefits observed in COVID-19. Samples and data from the clinical trials and further observational or epidemiological studies will be valuable in determining the extent of any clinical significance in protection from COVID-19.

Collectively, the tested parameters indicate that BCG-primed T cells are able to produce superior effector functions upon SARS-CoV-2-peptide restimulation compared to those not presensitized with BCG peptides. The observed cross-reactive effects of CD4+ and CD8+ T cells may be of importance in swift and effective viral clearance of SARS-CoV-2, reducing the severity of symptoms in COVID-19 patients pre-vaccinated with BCG. However, the *in vivo* effect of these cross-reactive T cells in BCG vaccinated individuals, especially memory T cell subsets remains to be determined.

The continuation of the more than 30 clinical trials assessing the role of BCG in COVID-19 is necessary to further gain insights into the extent of the protective effect. Once enough data is available about the extent of the protective effect BCG could have in COVID-19, a decision about its adoption as a vaccination in lieu of a specific COVID-19 vaccine could lead to improved patient outcomes. This is particularly important in settings where COVID-19 vaccine availability remains scarce or non-existent and in high-risk groups such as health care workers and the elderly.

## Conclusions

Collectively, our results demonstrate CD4+ and CD8+ T cells specific for BCG peptides cross-react with SARS-CoV-2 peptides. These data provide a possible mechanistic explanation for the observed negative epidemiological associations between BCG vaccinations and COVID-19 severity and mortality and support the continuation of clinical trials around the world, particularly in people at high-risk of contracting SARS-CoV-2 or with a heightened risk of COVID-19 mortality.

## Data Availability Statement

The original contributions presented in the study are included in the article/**Supplementary Material**. Further inquiries can be directed to the corresponding author.

## Ethics Statement

The studies involving human participants were reviewed and approved by Monash University Human Research Ethics Committee. The patients/participants provided their written informed consent to participate in this study.

## Author Contributions 

PE designed and performed research, analyzed data, and wrote the manuscript. BN performed experiments and analyzed data. JC and AF performed experiments. WW analyzed data. RC, P-YG, and SH analyzed data and provided intellectual input. JO designed research, analyzed data, and wrote the manuscript. All authors contributed to the article and approved the submitted version.

## Funding

This work was supported by Monash Health Foundation COVID-19 Research Fund Grant. The funders had no role in study design, data collection and analysis, decision to publish, or preparation of the manuscript.

## Conflict of Interest

The authors declare that the research was conducted in the absence of any commercial or financial relationships that could be construed as a potential conflict of interest.

## Publisher’s Note

All claims expressed in this article are solely those of the authors and do not necessarily represent those of their affiliated organizations, or those of the publisher, the editors and the reviewers. Any product that may be evaluated in this article, or claim that may be made by its manufacturer, is not guaranteed or endorsed by the publisher.
