# Supplementary material for: BCG Vaccine Derived Peptides Induce SARS-CoV-2 T Cell Cross-Reactivity
Source: Front Immunol. 2021 Aug 5;12:692729. doi: 10.3389/fimmu.2021.692729 (PMC8374943; doi:10.3389/fimmu.2021.692729)
Supplement: Supplementary file 1 [file DataSheet_1.pdf]

Supplementary Material

1 Supplementary Figures and Tables

Supplementary tables

Table S1a | Sequence homology between BCG and SARS-CoV-2 15mers.

Amino acid sequence alignment of the 15mer peptide pairs (PP) of BCG and SARS-CoV-2 used in this study including NCBI accession number, similarity, identity and BLOSUM62 matrix score. | - an identical amino acid match, : - a similar amino acid match, . – no match. RBN - RecB nuclease, MDCP - macro domain containing protein, UPF - UPF0189 protein, ZMP - zinc metalloprotease FtsH.

<table>
<tr><th>Peptide Pair</th><th>Accession / Protein name / Organism</th><th>Alignment</th><th>Identity</th><th>Similarity</th><th>Score</th></tr>
<tr><td rowspan="3">PP 1</td><td>KAF3412556.1 / RBN / M. bovis</td><td>759- S S Y L A V H G P P G T G K T -773</td><td rowspan="3">8/15 (53.3%)</td><td rowspan="3">10/15 (66.7%)</td><td rowspan="3">49.0</td></tr>
<tr><td></td><td>. . | . . : . | | | | | | | :</td></tr>
<tr><td>YP_009725308.1 / NSP13 / SARS-CoV-2</td><td>275- Q K Y S T L Q G P P G T G K S -289</td></tr>
<tr><td rowspan="3">PP 2</td><td>KAF3412556.1 / RBN / M. bovis</td><td>766- G P P G T G K T Y T A A R V I -780</td><td rowspan="3">8/15 (53.3%)</td><td rowspan="3">11/15 (73.3%)</td><td rowspan="3">44.0</td></tr>
<tr><td></td><td>| | | | | | | : : . | . . : .</td></tr>
<tr><td>YP_009725308.1 / NSP13 / SARS-CoV-2</td><td>282- G P P G T G K S H F A I G L A -296</td></tr>
<tr><td rowspan="3">PP 3</td><td>KAF3412556.1 / RBN / M. bovis</td><td>952- V S A L S Y E G R L C S H T E -966</td><td rowspan="3">7/15 (46.7%)</td><td rowspan="3">11/15 (73.3%)</td><td rowspan="3">36.0</td></tr>
<tr><td></td><td>| | | | . | : . : | . : | . :</td></tr>
<tr><td>YP_009725308.1 / NSP13 / SARS-CoV-2</td><td>452- V S A L V Y D N K L K A H K D -466</td></tr>
<tr><td rowspan="3">PP 4</td><td>KAF3412556.1 / RBN / M. bovis</td><td>1093- R N R L N V A V S R A Q Y A A -1107</td><td rowspan="3">7/15 (46.7%)</td><td rowspan="3">10/15 (66.7%)</td><td rowspan="3">34.0</td></tr>
<tr><td></td><td>. | | . | | | : : | | : . . .</td></tr>
<tr><td>YP_009725308.1 / NSP13 / SARS-CoV-2</td><td>558- V N R F N V A I T R A K V G I -572</td></tr>
<tr><td rowspan="3">PP 5</td><td>WP_003909539.1 / MDCP / M. bovis</td><td>214- L R H A G G V A A A I A R A G -228</td><td rowspan="3">8/15 (53.3%)</td><td rowspan="3">11/15 (73.3%)</td><td rowspan="3">42.0</td></tr>
<tr><td></td><td>| : | . | | | | . | : . : | .</td></tr>
<tr><td>YP_009725299.1 / NSP3 / SARS-CoV-2</td><td>247- L K H G G G V A G A L N K A T -261</td></tr>
<tr><td rowspan="3">PP 6</td><td>AHM07651.1 / UPF / M. bovis</td><td>25- A N T R L R H A G G V A A A I -39</td><td rowspan="3">9/15 (60.0%)</td><td rowspan="3">11/15 (73.3%)</td><td rowspan="3">48.0</td></tr>
<tr><td></td><td>| | . . | : | . | | | | . | :</td></tr>
<tr><td>YP_009725299.1 / NSP3 / SARS-CoV-2</td><td>243- A N V Y L K H G G G V A G A L -257</td></tr>
<tr><td rowspan="3">PP 7</td><td>AHM07651.1 / UPF / M. bovis</td><td>28- R L R H A G G V A A A I A R A -42</td><td rowspan="3">8/15 (53.3%)</td><td rowspan="3">11/15 (73.3%)</td><td rowspan="3">42.0</td></tr>
<tr><td></td><td>. | : | . | | | | . | : . : |</td></tr>
<tr><td>YP_009725299.1 / NSP3 / SARS-CoV-2</td><td>246- Y L K H G G G V A G A L N K A -260</td></tr>
<tr><td rowspan="3">PP 8</td><td>AMC52863.1 / ZMP / M. bovis</td><td>71- L Y G P P G T G K T L L A R A -95</td><td rowspan="3">9/15 (60.0%)</td><td rowspan="3">10/15 (66.7%)</td><td rowspan="3">44.0</td></tr>
<tr><td></td><td>| . | | | | | | | : . . | . .</td></tr>
<tr><td>YP_009725308.1 / NSP13 / SARS-CoV-2</td><td>280- L Q G P P G T G K S H F A I G -294</td></tr>
</table>

Table S1b | Sequence homology between BCG and SARS-CoV-2 9mers

Amino acid sequences of the 9mer peptide pairs (PP) of BCG and SARS-CoV-2 used in this study. The 9mers constitute the 15mers described in Table S1a. Percent similarity is shown.

Peptide pair	BCG peptide	SARS-CoV-2 peptide	% Similarity	Peptide pair	BCG peptide	SARS-CoV-2 peptide	% Similarity
PP1	SSYLAVHGP	QKYSTLQGP	44.4	PP5	LRHAGGVAA	LKHGGGVAG	77.8
	SYLAVHGPP	KYSTLQGPP	55.6		RHAGGVAAA	KHGGGVAGA	77.8
	YLAVHGPPG	YSTLQGPPG	66.7		HAGGVAAAI	HGGGVAGAL	77.8
	LAVHGPPGT	STLQGPPGT	66.7		AGGVAAAIA	GGGVAGALN	66.7
	AVHGPPGTG	TLQGPPGTG	77.8		GGVAAAIAR	GGVAGALNK	77.8
	VHGPPGTGK	LQGPPGTGK	88.9		GVAAAIARA	GVAGALNKA	77.8
	HGPPGTGKT	QGPPGTGKS	88.9		VAAAIARAG	VAGALNKAT	66.7
PP2	GPPGTGKTY	GPPGTGKSH	100	PP6	ANTRLRHAG	ANVYLKHGG	66.7
	PPGTGKTYT	PPGTGKSHF	88.9		NTRLRHAGG	NVYLKHGGG	66.7
	PGTGKTYTA	PGTGKSHFA	88.9		TRLRHAGGV	VYLKHGGGV	66.7
	GTGKTYTAA	GTGKSHFAI	77.8		RLRHAGGVA	YLKHGGGVA	77.8
	TGKTYTAAR	TGKSHFAIG	66.7		LRHAGGVAA	LKHGGGVAG	77.8
	GKTYTAARV	GKSHFAIGL	66.7		RHAGGVAAA	KHGGGVAGA	77.8
	KTYTAARVI	KSHFAIGLA	55.6		HAGGVAAAI	HGGGVAGAL	77.8
PP3	VSALSYEGR	VSALVYDNK	77.8	PP7	RLRHAGGVA	YLKHGGGVA	77.8
	SALSYEGRL	SALVYDNKL	77.8		LRHAGGVAA	LKHGGGVAG	77.8
	ALSYEGRLC	ALVYDNKLK	66.7		RHAGGVAAA	KHGGGVAGA	77.8
	LSYEGRLCS	LVYDNKLKA	66.7		HAGGVAAAI	HGGGVAGAL	77.8
	SYEGRLCSH	VYDNKLKAH	66.7		AGGVAAAIA	GGGVAGALN	66.7
	YEGRLCSHT	YDNKLKAHK	66.7		GGVAAAIAR	GGVAGALNK	77.8
	EGRLCSHTE	DNKLKAHKD	66.7		GVAAAIARA	GVAGALNKA	77.8
PP4	RNRLNVAVS	VNRFNVAIT	77.8	PP8	LYGPPGTGK	LQGPPGTGK	88.9
	NRLNVAVSR	NRFNVAITR	88.9		YGPPGTGKT	QGPPGTGKS	88.9
	RLNVAVSRA	RFNVAITRA	88.9		GPPGTGKTL	GPPGTGKSH	88.9
	LNVAVSRAQ	FNVAITRAK	88.9		PPGTGKTLL	PPGTGKSHF	77.8
	NVAVSRAQY	NVAITRAKV	88.9		PGTGKTLLA	PGTGKSHFA	77.8
	VAVSRAQYA	VAITRAKVG	77.8		GTGKTLLAR	GTGKSHFAI	66.7
	AVSRAQYAA	AITRAKVGI	66.7		TGKTLLARA	TGKSHFAIG	55.6

Table S2a| Donor characteristics

**Number:**	**20**
**Age:**	**21-42**
**Gender:**	
	**Male: 50% (10/20)**
	**Female: 50% (10/20)**
**HLA typing:**	
	**High resolution HLA-typed: 35% (7/20)**
	**HLA-DR typed: 15% (3/20)**
	**Not HLA-typed: 50% (10/20)**
**Sars-CoV-2 status:**	
	**IgG positivity: 0% (0/20)**
	**IgM positivity: 0% (0/20)**

Table S2b| Donor HLA alleles used in this study.

Donor ID	HLA-A	HLA-B	HLA-C	HLA-DRB1	HLA-DRB3	HLA-DRB4	DPB1	DPA1	DQB1	DQA1
H1	11:01, 33:03	58:01	03;02	03:01, 04:05	02:0,	01:03	04:01, 05:01	01:03, 02:02	02:01, 04:01	03:03, 05:01
H2	01:01, 32:01	14:01, 44;02	05:01, 08:02	07:01, 11:01	02:02	01:01	04:02	01:03	02:02, 03:01	02:01, 05:05
H3	02:07, 32:01	48:01, 52:01	08:03, 12:02	09:01, 14:05	02:02:01G	01:03	02:01, 02:02	01:03, 02:02	03:03, 05:03	01:04, 03;02
H4	02:01, 11:01	14:02, 35:01	04:01, 08:02	01:02, 14:54	02:02		04:01, 16:01	01:03	05:01, 05:03	01:01, 01:04
H6				03,04						
H7	03:01, 11:01	38:01, 54:01	07:02, 12:03	04:05, 07:01		01:03	02:02:01G, 05:01:01G	02:02	02:02/156/163N, 04:01	02:01, 03:03
H8	11:01	13:01, 40:01	03:04, 07:02	08:09, 12:02	03:01		03:01, 05:01	01:03, 02:02	03:01, 04:02	04:01, 06:01
H9				09,16						
H11				12						
H14	30:01, 31:01	13:02, 40:01	03:04, 06:02	07:01, 12:01/10	01:01	01:03	05:01	02:02	02:02/156/163N, 03:01/276N	02:01, 05:08

Supplementary figures:

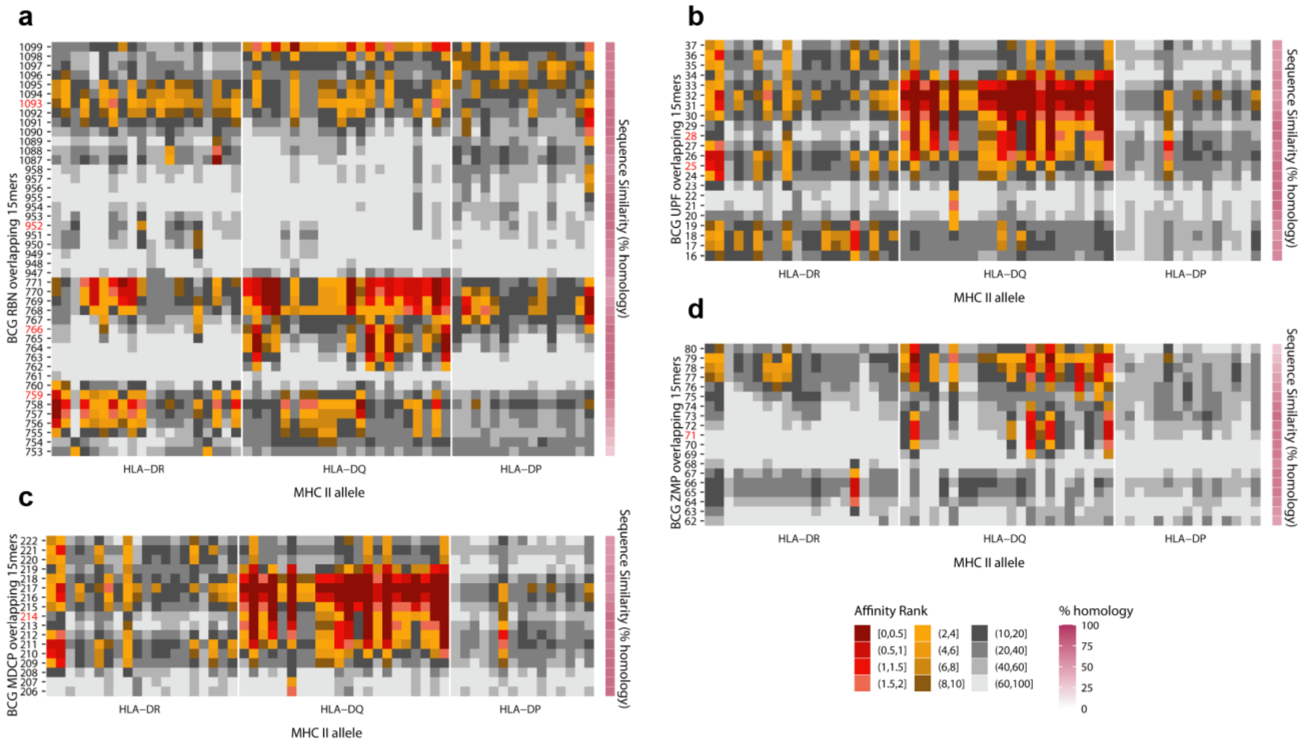

Figure S1| Regions of BCG-SARS-CoV-2 homology exhibit broad HLAII binding

Affinity rank score of BCG 15mers overlapping by 1 amino acid across the region of shared homology between BCG and SARS-CoV-2. Hotspots of high affinity overlap broadly with high regions of homology. Red gradient; strong peptide-MHC binder (affinity rank $\leq$ 2). Yellow gradient; peptide-MHC binder (affinity rank $>$ 2 and $\leq$ 10). Grey gradient; non-binder (affinity rank $<$ 10). Y-axis; number indicates the amino acid sequence start number of the respective 15mer. Red number indicates the 15mers analysed in this study. X axis; MHC class II alleles grouped into HLA-DR (n=20), HLA-DQ (n=22) and HLA-DP (n=15) isotype. The selected alleles are globally representative and include all alleles from HLA-typed donors used in this study. Pink gradient; Pairwise percent sequence similarity between BCG and SARS-CoV-2 15mers. a) BCG RecB nuclease (RBN) affinity rank binding scores. b) BCG UPF0189 protein (UPF) affinity rank binding scores. c) BCG macro domain containing protein (MDCP) affinity rank binding scores. d) BCG zinc metalloprotease FtsH (ZMP) affinity rank binding scores.

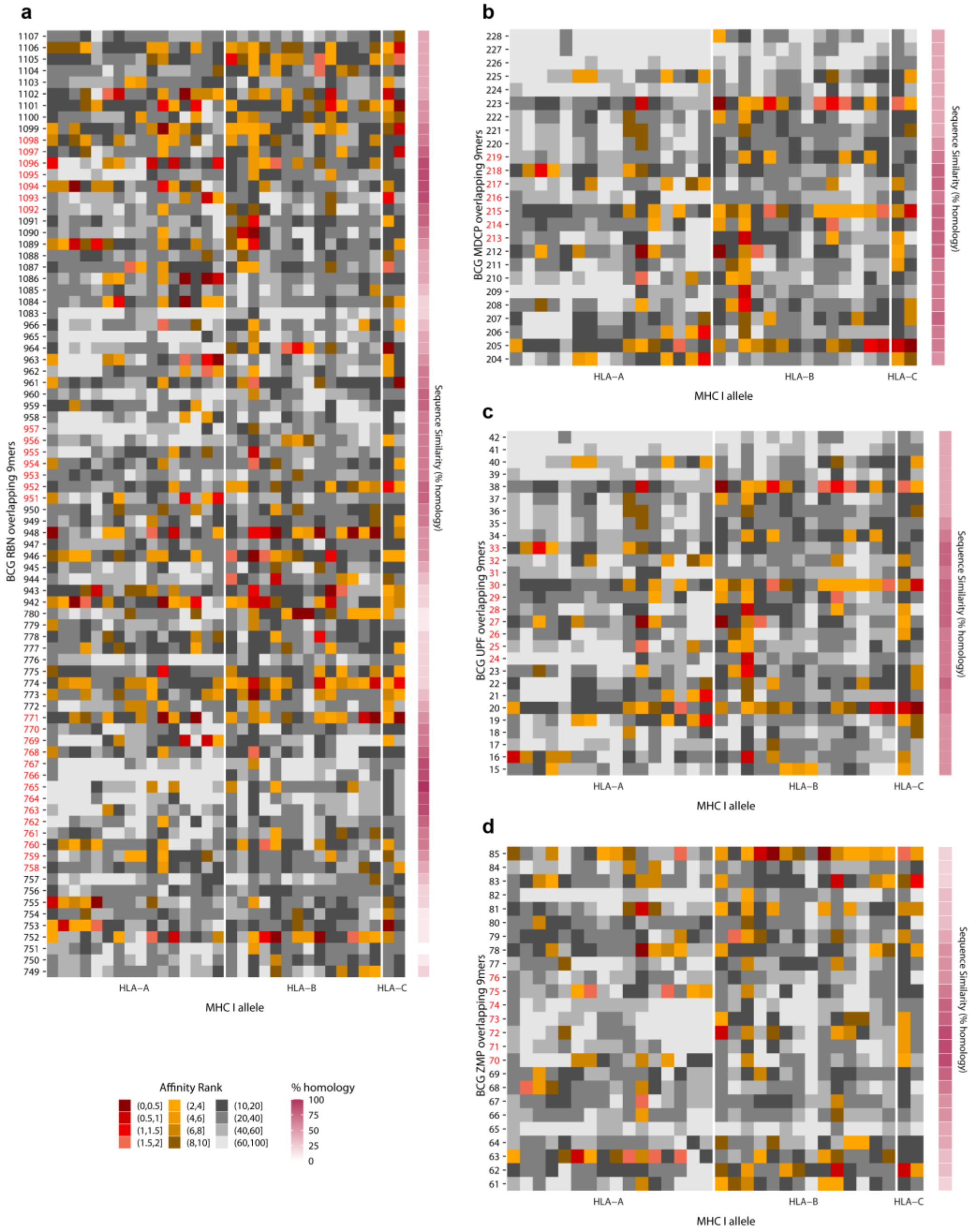

Figure S2| Regions of BCG-SARS-CoV-2 homology exhibit broad HLA-I binding

Affinity rank score of BCG 9mers overlapping by 1 amino acid across the region of shared homology between BCG and SARS-CoV-2. Red gradient; strong peptide-MHC binder (affinity rank $\leq$ 2). Yellow gradient; peptide-MHC binder (affinity rank $>$ 2 and $\leq$ 10). Grey gradient; non-binder (affinity rank $<$ 10). Y-axis; number indicates the amino acid sequence start number of the respective 9mer. Red number indicates the overlapping 9mers contained within the 15mers analysed in this study. X-axis; MHC class I alleles grouped into HLA-A (n=16), HLA-B (n=14) and HLA-C (n=2) isotype. These

alleles selected are globally representative and include the alleles from HLA-typed donors used in this study. Pink gradient; Pairwise percent sequence similarity between BCG and SARS-CoV-2 9mers. a) BCG RecB nuclease (RBN) affinity rank binding scores. b) BCG macro domain containing protein (MDCP) affinity rank binding scores. c) BCG UPF0189 protein (UPF) affinity rank binding scores. d) BCG zinc metalloprotease FtsH (ZMP) affinity rank binding scores.

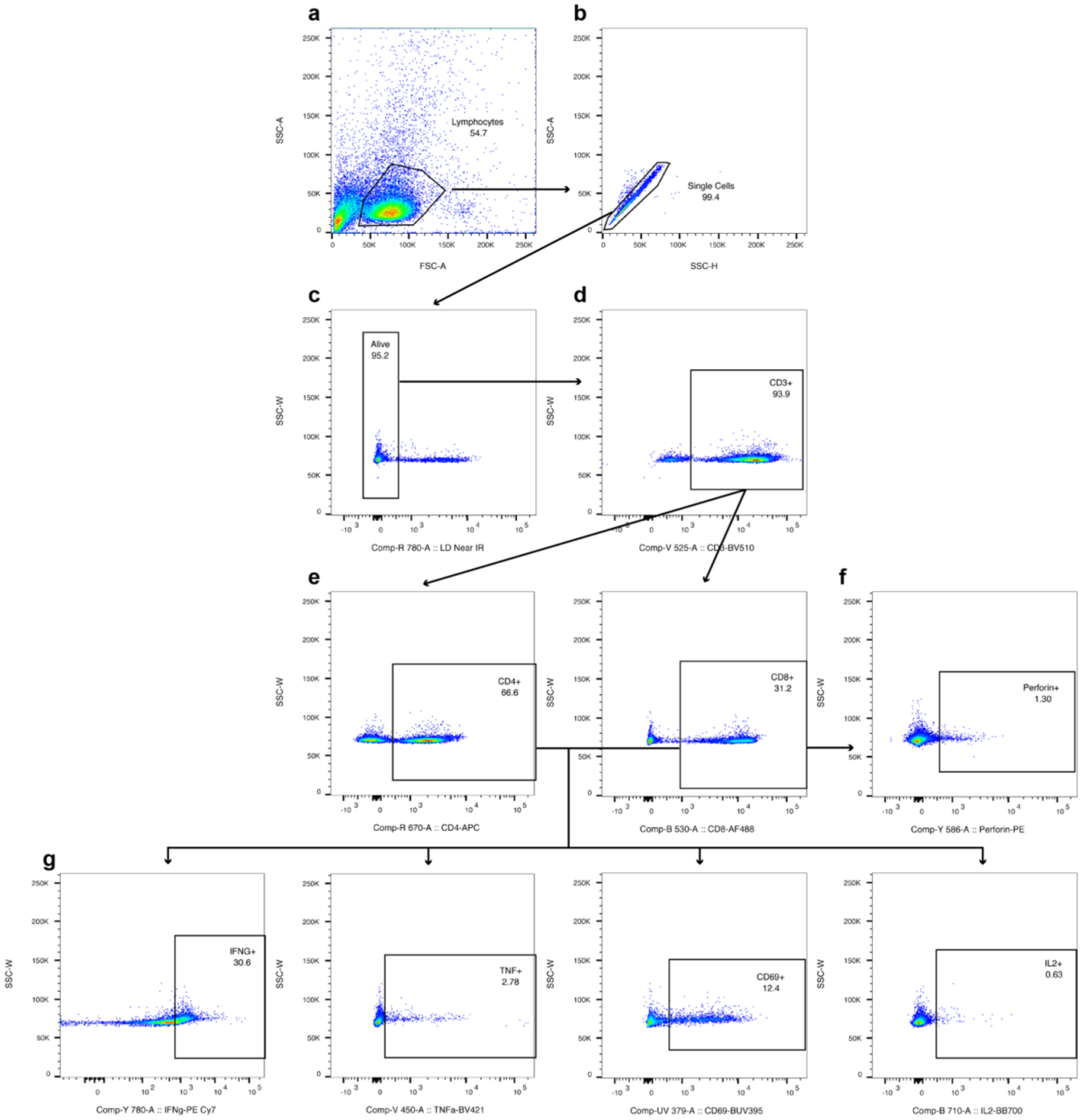

Figure S3: Flow Cytometry Gating Strategy for intracellular cytokine staining

Intracellular Cytokine Staining (ICS) panel. a) Forward scatter area (FSC-A) versus side scatter area (SSC-A) density plot gating the lymphocyte population. b) Side scatter height (SSC-H) versus SSC-A density plot gating the single cell population. c) Live/dead discrimination dye (LD Near IR) versus side scatter width (SSC-W) density plot gating the alive cell population. d) CD3 versus SSC-W density plot gating the CD3+ T cells. e) CD3+ T cells are separated into CD4+ and CD8+. f) Perforin positive gating is generated from the CD8+ parent population. g) IGNG, TNF, CD69 and IL-2 positive gates are generated from the CD4+ and CD8+ parent population. All fluorescence based gating is determined based on fluorescence minus one (FMO) controls.

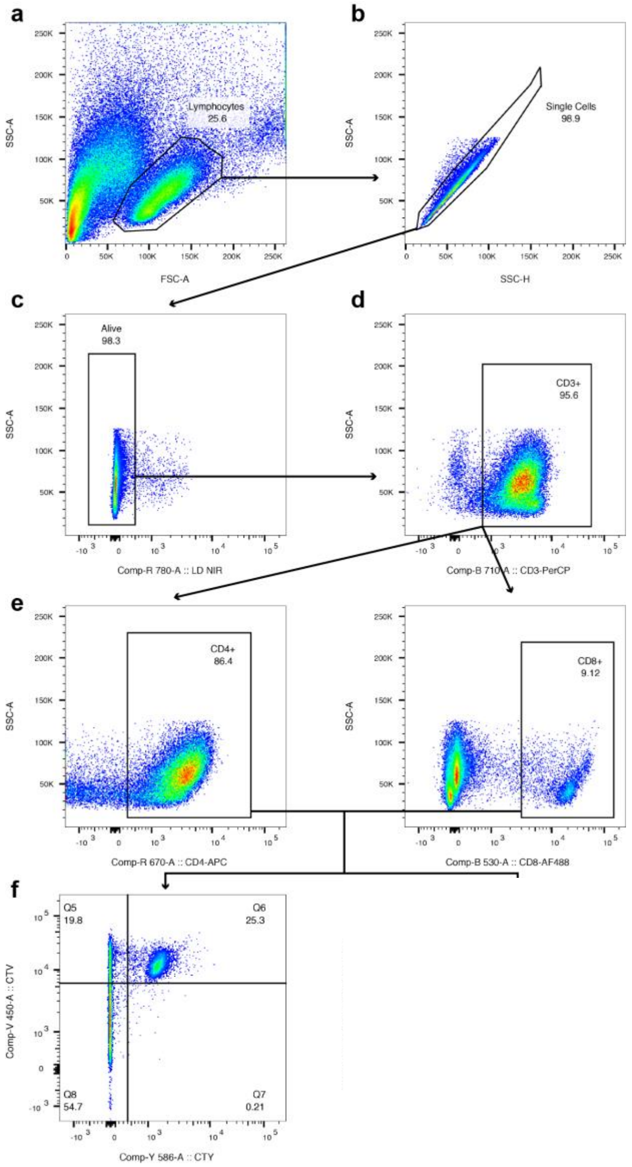

Figure S4: Flow Cytometry Gating Strategy for T cell proliferation

T cell proliferation and memory gating strategy. a) Forward scatter area (FSC-A) versus side scatter area (SSC-A) density plot gating the lymphocyte population. b) Side scatter height (SSC-H) versus SSC-A density plot gating the single cell population. c) Live dead discrimination dye (LD Near IR) versus SSC-A density plot gating the alive cell population. d) CD3 versus SSC-A density plot gating the CD3+ T cells. e) The $CD3^{+}$ cells are separated into $CD4^{+}$ and CD8+ T cells. f) The proliferation dyes Cell Trace Yellow (CTY) versus Cell Trace Violet (CTV) density plots are quadrant gated based on proliferation after priming and restimulation. Q5 – CTY^{low} CTV^{high} T cells that proliferated after priming but not after restimulation. Q6 – CTY^{high} CTV^{high} T cells that did not proliferate after priming or restimulation. Q7 CTY^{high} CTV^{low} T cells that did not proliferate after priming but proliferated after restimulation. Q8 – CTY^{low} CTV^{low} T cells that proliferated both after priming and restimulation. Fluorescence minus one (FMO) controls were used to determine fluorescence based gating except CTY and CTV, which are based on the point at which the first cell division took place visible by fluorescence dye dilution.

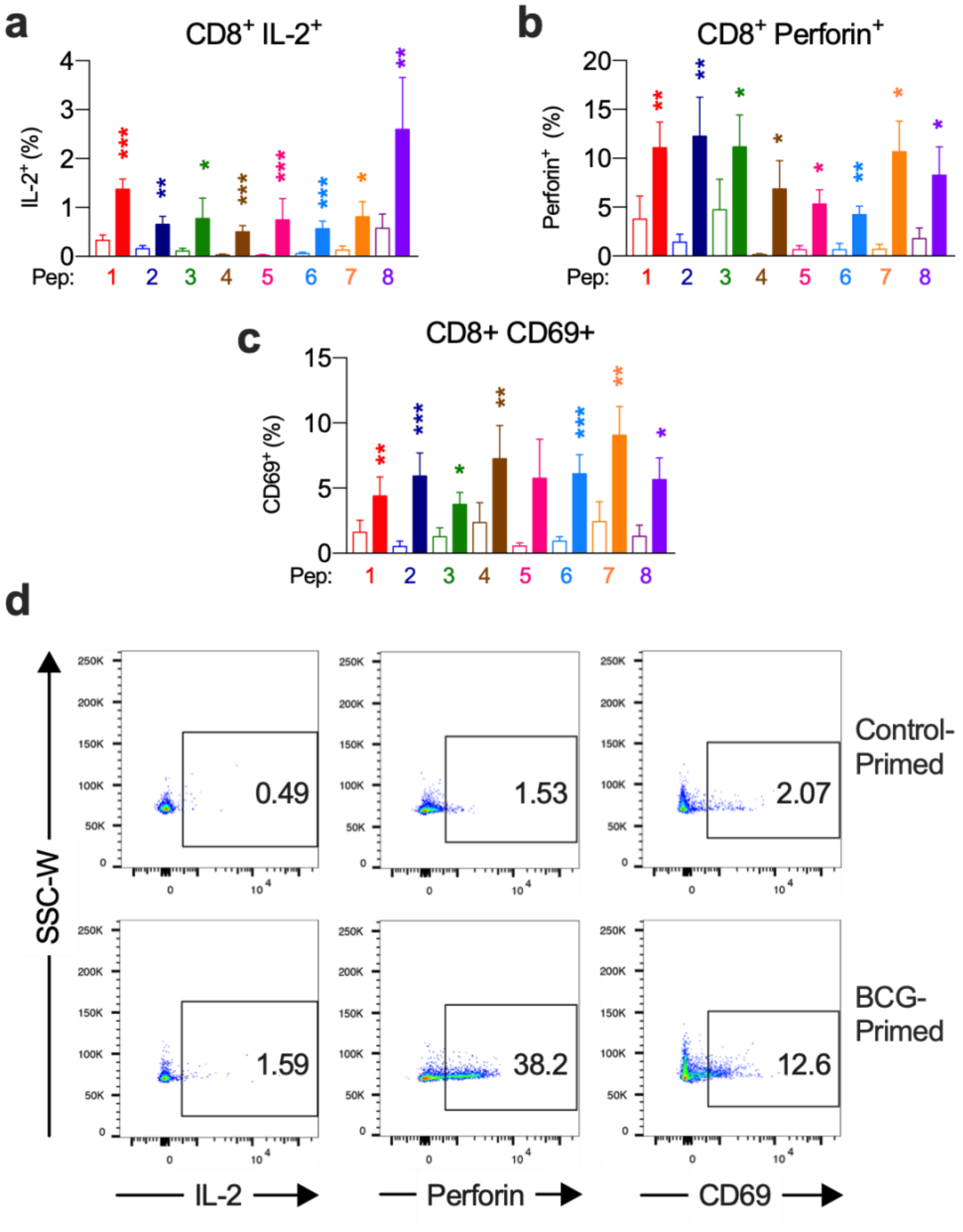

Figure S5| BCG priming enhances IL-2, Perforin and CD69 CD8 T cell responses against SARS-CoV-2

BCG-peptide 1-8 primed (shaded bars) or control primed using irrelevant peptide (unshaded bars) $CD3^+$ T cells were restimulated with SARS-CoV-2-peptide-homologue-pulsed dendritic cells for 6hr and analysed by intracellular cytokine staining. Responders were selected as per methods section for analysis. *$P < 0.05$, **$P < 0.01$, ***$P < 0.001$ by Wilcoxon matched-pairs signed rank test. a) $CD8^+$ $IL\text{-}2^+$ responses (n=7-13). b) $CD8^+$ $Perforin^+$ responses (n=6-14). c) $CD8^+$ $CD69^+$ responses (n=5-12). d) Representative IL-2, perforin and CD69 dot plots of a responder control-primed (top) and BCG-primed (bottom) donor.

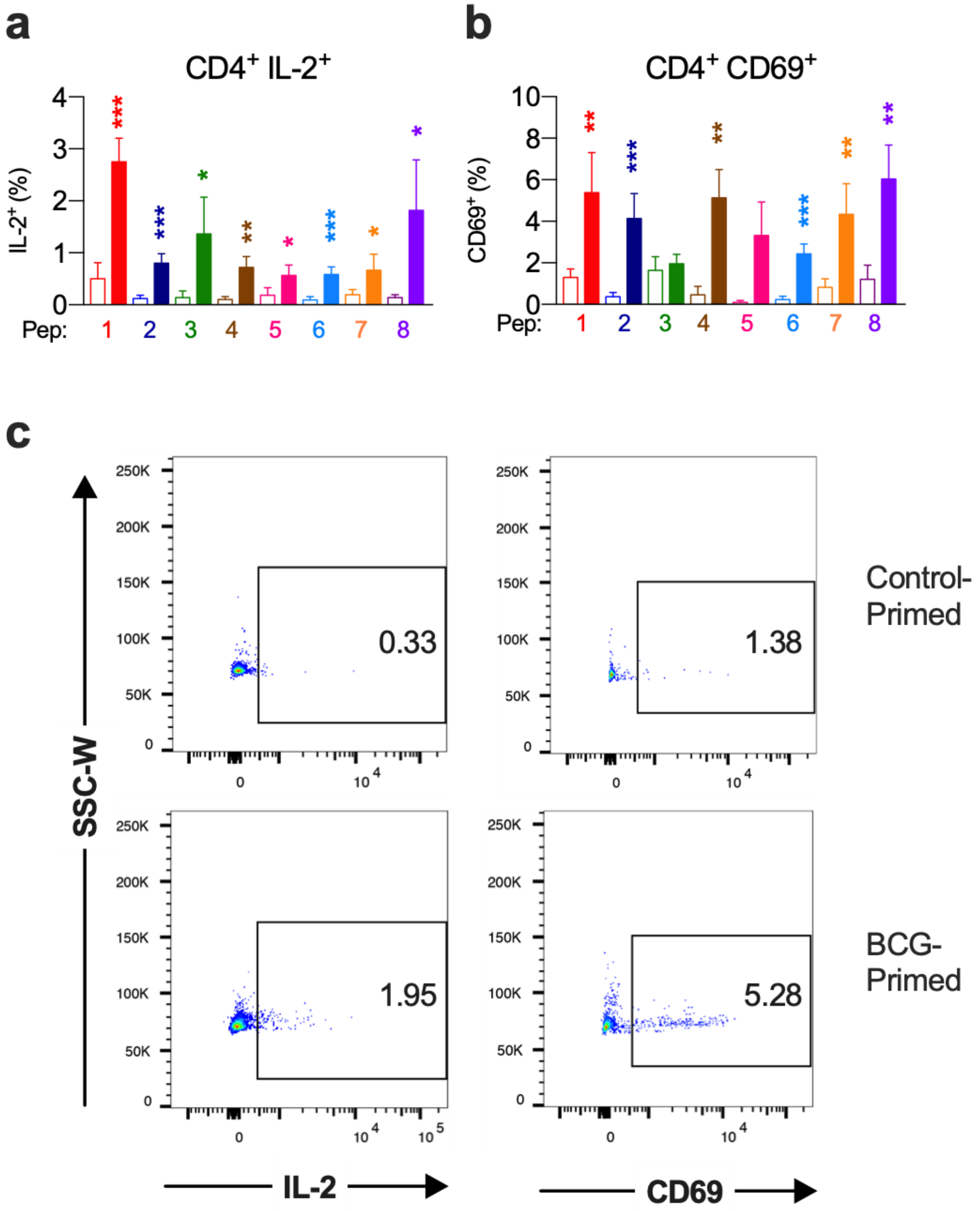

Figure S6| BCG priming enhances IL-2 and CD69 CD4 T cell responses against SARS-CoV-2

BCG-peptide 1-8 primed (shaded bars) or control primed using irrelevant peptide (unshaded bars) $CD3^+$ T cells were restimulated with SARS-CoV-2-peptide-homologue-pulsed dendritic cells for 6hr and analysed by intracellular cytokine staining. Responders were selected as per methods section. $*P < 0.05$, $**P < 0.01$, $***P < 0.001$ by Wilcoxon matched-pairs signed rank test. a) $CD4^+$ $IL\text{-}2^+$ responses (n=7-13). b) $CD4^+$ $CD69^+$ responses (n=8-13). c) Representative IL-2 and CD69 dot plots of a responder control-primed (top) and BCG-primed (bottom) donor.

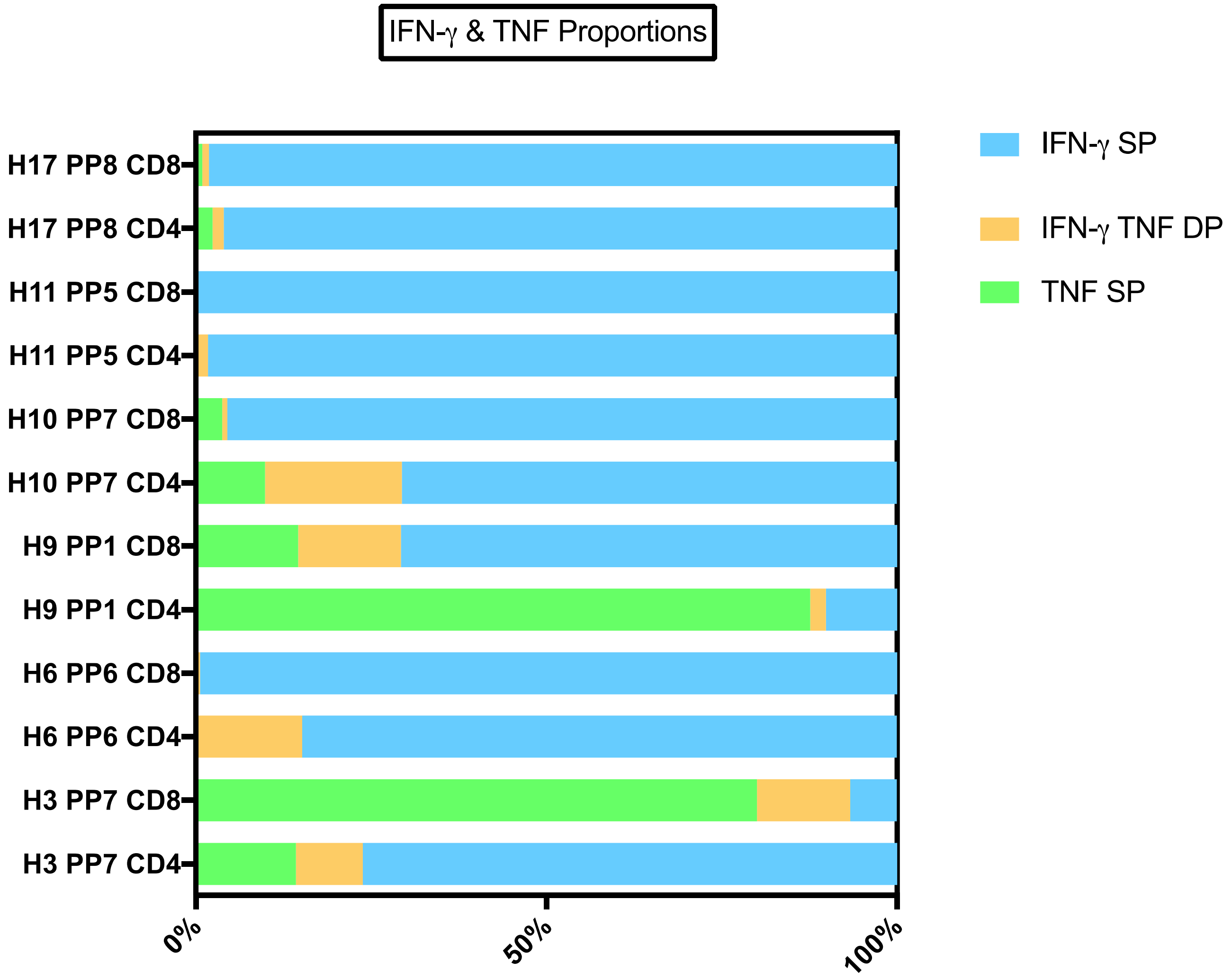

Figure S7| TNF IFN-γ proportions of responder individuals.

BCG-primed individuals who responded to SARS-CoV-2-peptide restimulation mostly exhibited a strong IFN-γ signature with a lower proportion of TNF, although some individuals showed a dominant TNF response. Across individuals and peptide pairs, a variable proportion of IFN-γ SP, TNF SP and IFN-γ, TNF DP was observed. A selection of 6 individuals CD4+ and CD8+ responses (of N = 20 tested) against 5 peptide pairs (PP) of 8 peptide pairs tested. IFN-γ single positive (SP) – proportion of cells producing IFN-γ and not TNF. TNF SP – proportion of cells producing TNF and not IFN-γ. IFN-γ TNF double positive (DP) – proportion of cells producing TNF and not IFN-γ.

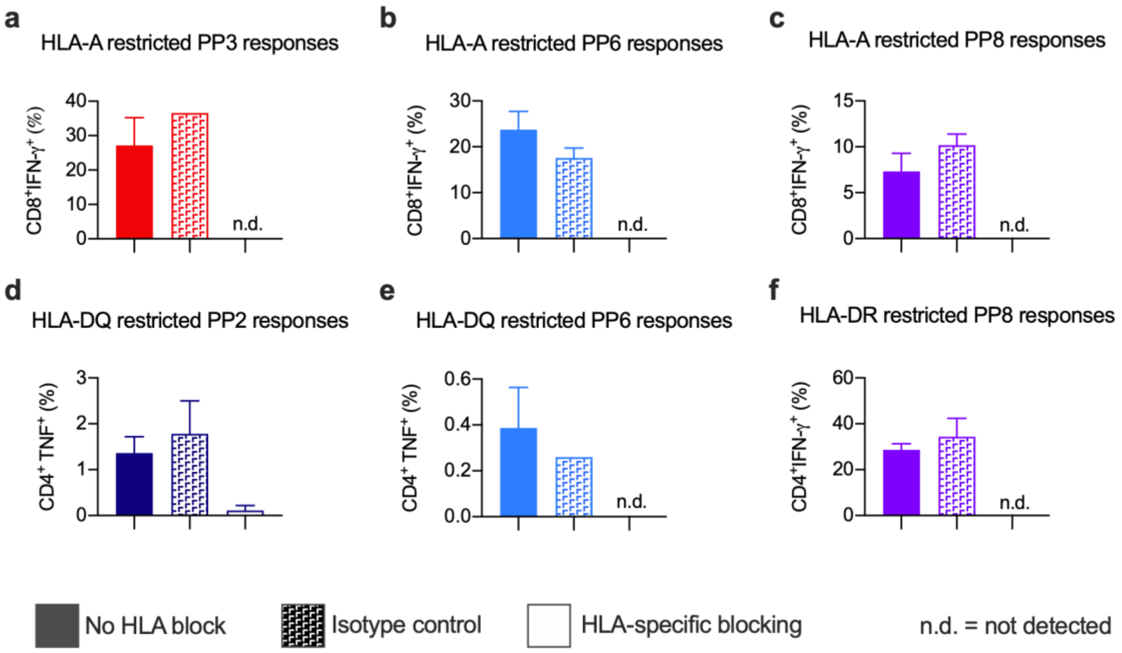

Figure S8| HLA blocking abrogates T cell responses

To confirm that the T cell responses observed were mediated by presentation of peptides by specific HLA we used HLA-specific blocking antibodies to block specific HLA. Blocking of the MHC class I or II allele predicted to bind the SARS-CoV-2 restimulation peptide almost completely abrogates T cell responses in CD8+ T cells for MHC class I blocking and CD4+ T cells for MHC class II blocking. This indicates that T cell receptor binding to peptide-MHC is responsible for the T cell response to SARS-CoV-2 peptide restimulation. Three HLA-typed donor (H1, H2 and H14) responses expressing the appropriate HLA were tested.

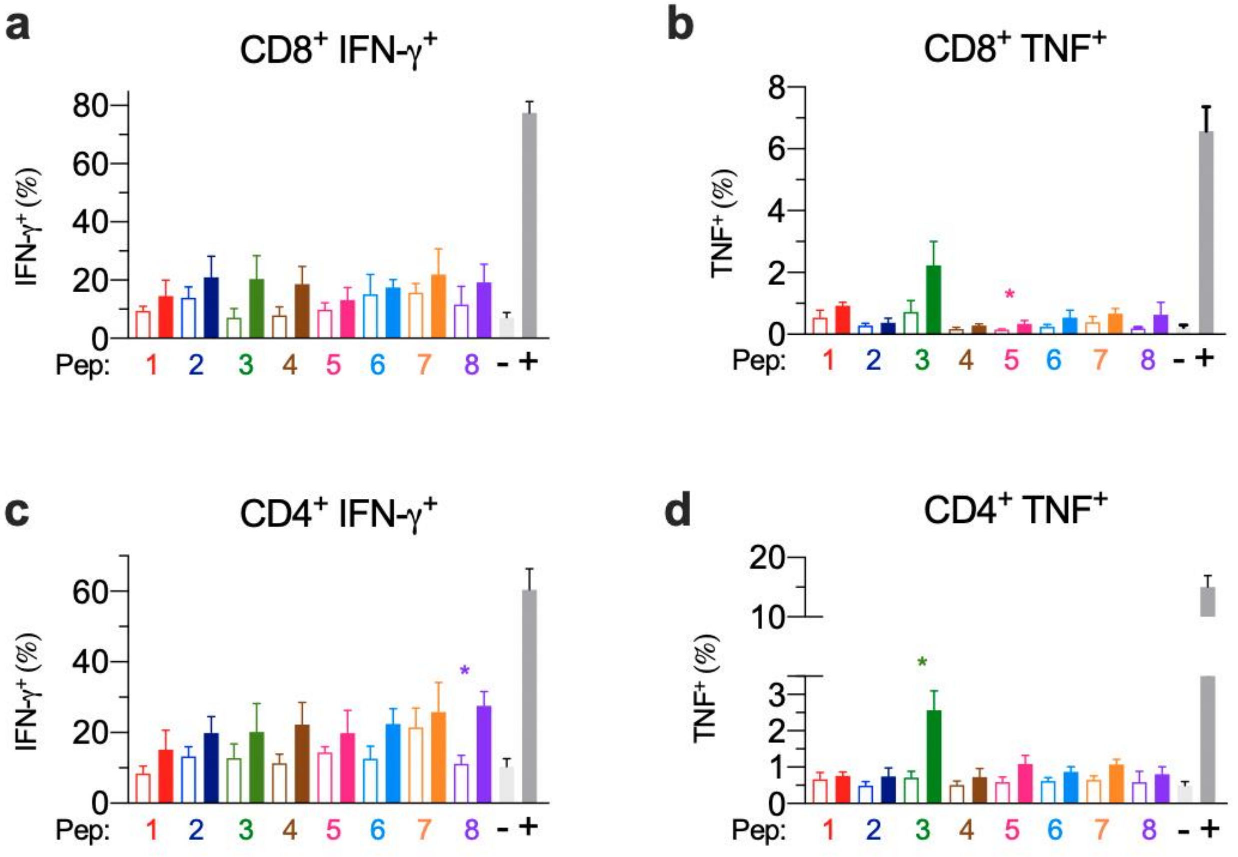

Figure S9| BCG vaccinated individuals exhibit increased SARS-CoV-2 T cell responses

Individuals vaccinated with BCG (n=9, shaded bars) exhibited increased responses to SARS-CoV-2 peptides 1-8 compared to individuals not vaccinated with BCG (n=6, unshaded bars). $CD3^+$ T cells were cultured with immature DCs pulsed with one of the 8 SARS-CoV-2 peptides from Fig. 1 for 6hr and cytokine responses were measured by intracellular cytokine staining. a) $CD8^+$ IFN-γ^+ responses (n=4-7), b) $CD8^+$ TNF^+ responses (n=4-8), c) $CD4^+$ IFN-γ^+ responses (n=4-5), d) $CD4^+$ TNF^+ responses (n=4-9). *$P < 0.05$, by two-tailed, Mann-Whitney test. + are positive co-culture stimulated with anti-CD3, anti-CD28 microbeads. – are negative control co-culture not stimulated with peptide or microbeads.
